# Retraction Note: MicroRNA-93-5p promotes epithelial-mesenchymal transition in gastric cancer by repressing tumor suppressor AHNAK expression

**DOI:** 10.1186/s12935-024-03340-2

**Published:** 2024-04-30

**Authors:** Erdong Shen, Xin Wang, Xin Liu, Mingyue Lv, Liang Zhang, Guolian Zhu, Zhe Sun

**Affiliations:** 1 Department of Surgical Oncology and General Surgery, Key Laboratory of Precision Diagnosis and Treatment of Gastrointestinal Tumors, Ministry of Education, The First Affiliated Hospital of China Medical University, No. 155, Nanjing North Road, Heping District, Shenyang, 110001 Liaoning People’s Republic of China; 2 Department of Oncology, Yueyang First People’s Hospital, Yueyang, 414000 P. R. China; 3 Department of Thoracic Surgery, Cancer Hospital of China Medical University/Liaoning Cancer Hospital, 110001 Shenyang, P. R. China; 4 Department of Oncology, Shenyang Fifth People Hospital, 110001 Shenyang, P. R. China

**Cancer Cell International (2020) 20:76**

10.1186/s12935-019-1092-7

The Editors have retracted this article at the corresponding author’s request. After the publication of this article, concerns have been raised regarding image overlaps. The authors, Zhe Sun, Xin Wang, Mingyue Lv, Xin Liu, Liang Zheng, and Guolian Zhu stated that they were not aware of the submission of this article and they have not contributed to the study presented in this article.

The Editors therefore no longer have confidence in a study presented in this article.

- Image overlap between Fig. 3e, a and b from [1].
- Image overlap between Figs. 5d and 6G of [2].
- Irregularities in western blots presented in Figs. 3f and 4e/f, 5c/f and 6a.

Authors, Zhe Sun, Xin Wang, Mingyue Lv, Xin Liu, Liang Zheng, and Guolian Zhu agree with this retraction. Author, Erdong Shen did not respond to correspondence from the Publisher about this retraction.
